# Paradoxical hepatic tumor: Undifferentiated embryonal sarcoma of the liver

**DOI:** 10.4103/0971-3026.59760

**Published:** 2010-02

**Authors:** Kushaljit Singh Sodhi, Elhamy Bekhitt, Christian Rickert

**Affiliations:** Department of Medical Imaging, Royal Children's Hospital, Melbourne, Victoria 3052, Australia; 1 Department of Pathology, Royal Children's Hospital, Melbourne, Victoria 3052, Australia

**Keywords:** Liver, tumor, children, embryonal sarcoma, imaging

## Abstract

Undifferentiated embryonal sarcoma (UES) is a rare primary malignant tumor of the liver that typically presents in late childhood. We report a case of primary UES, which had a typical paradoxical appearance on different imaging modalities.

## Introduction

Undifferentiated embryonal sarcoma (UES) represents 9-15% of all hepatic tumors in children and is the third most common malignant hepatic tumor in children after hepatoblastoma and hepatocellular carcinoma. However, it is still a rare entity and, till date, less than 150 cases of UES have been reported.[1–2] Recent published data indicate that modern surgical procedures along with neoadjuvant or adjuvant chemotherapy can lead to an increase in survival rates. Early diagnosis and prompt therapy are thus important.[2]

We present the radiological findings of UES of the liver in a 7-year-old child. These cases can be diagnostically challenging because of the discordance between the appearances at CT scan/MRI and at USG. While CT scan and MRI demonstrated a cyst-like mass, USG revealed a solid-looking mass lesion. Familiarity with the paradoxical findings of UES of the liver is useful in the differential diagnosis of primary hepatic tumors in childhood.

## Case Report

A 7-year-old girl presented with a 2-week history of shoulder-tip pain, progressive fever and right upper quadrant pain. She had no history of vomiting, diarrhea or weight loss.

On examination, she was febrile and had a palpable, firm mass in the right upper quadrant. She had a temperature of 38°C, heart rate of 145/min and respiratory rate of 28/ min. Blood culture was negative and did not grow any organism. Serology for cytomegalovirus (CMV), Ebstein-Barr virus, hepatitis B surface antigen, hepatitis C and hydatid was negative. Serum α-fetoprotein levels of 0 and serum HCG <2 were recorded.

**Figure 1 (a,b):**
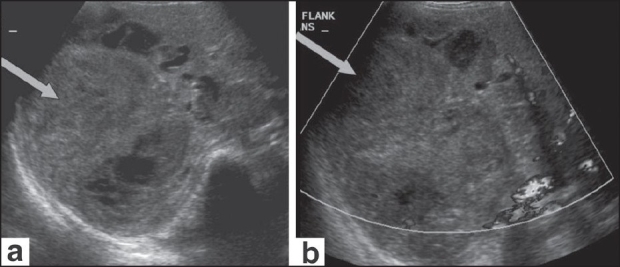
USG of the abdomen (a) reveals a large, complex, solid-cystic mass (arrow) within the right lobe of the liver, appearing predominantly solid in appearance. The color Doppler image (b) shows absence of increased vascularity in the mass lesion (arrows)

**Figure 2 (a,b):**
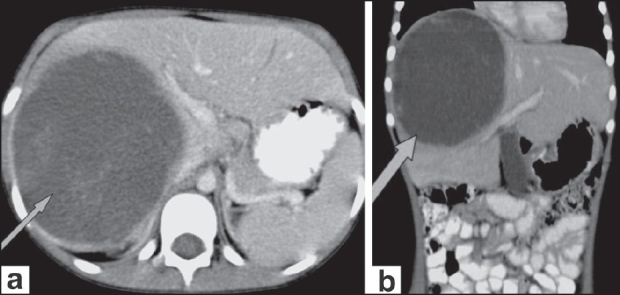
Axial (a) and coronal (b) images show a well-defined, solitary, predominantly cystic, mass lesion (arrows)

**Figure 3 (a-c):**
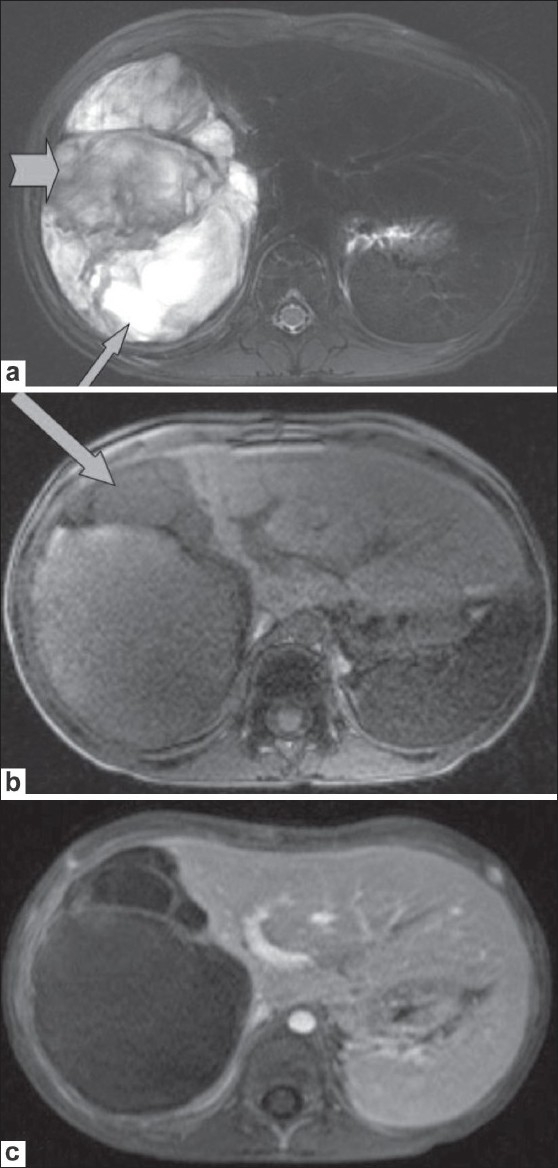
T2W axial MRI of the abdomen (a) shows the mass to be predominantly hyperintense (arrows), with hypointense areas (arrowheads) within. A T1W axial MRI image (b) shows the lesion to be iso to heterogeneously hypointense (arrows). A contrast-enhanced T1W axial MRI image (c) shows absence of enhancement

USG of the abdomen [Figure 1] showed a large, mixed, complex solid-cystic lesion, which was predominantly a solid lesion with a heterogenous cystic component, within the right lobe of the liver with no flow or increased vascularity on color Doppler examination. CT scan revealed a well-defined, solitary, cyst-like hepatic mass [Figure 2]. No definite solid mass-like component, enhancing nodule or calcification/fat was appreciated. A few hyperdense areas were seen within this cystic-looking lesion. No vascular compromise or invasion was seen. No contrast enhancement was seen. Subsequently, an MRI of the abdomen was also performed [Figure 3a], which revealed the hepatic lesion to be predominantly hyperintense on the T2W images, but with central areas of low signal intensity. The T1W axial images [Figure 3b] revealed the lesion to be iso to heterogeneously hypointense and did not show any enhancement after intravenous contrast administration [Figure 3c]. There was thus a discordance between the predominantly solid appearance on USG and the predominantly cystic appearance on CT scan and MRI.

**Figure 4:**
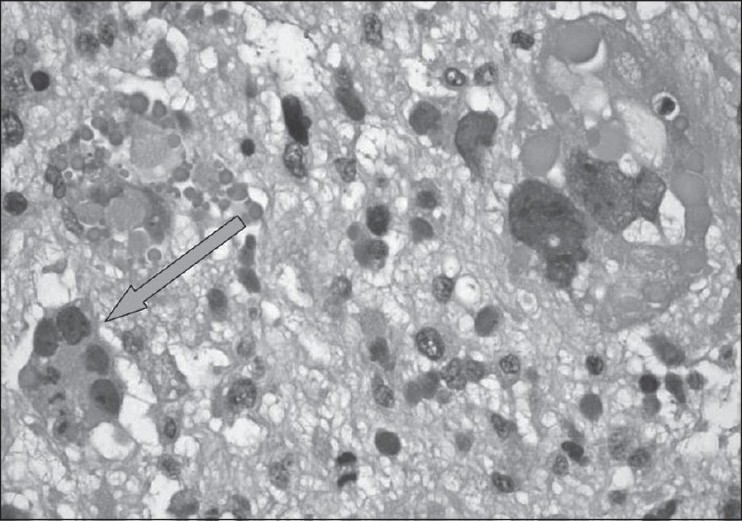
Photomicrograph (hematoxylin and eosin) shows a myxoid spindle-cell lesion with numerous bizarre and pleomorphic giant cells (arrow), mitoses and hyaline round globules

She underwent surgical biopsy. Immunohistochemistry was performed, which revealed that tumor cells were positive for α-1-antitrypsin and α-1-antichymotrypsin and some cells were also positive for cytokeratosis. No positivity was seen for CD10 or desmin. Several CD68-positive macrophages were found. The myxoid morphology of the lesions, the presence of eosinophilic hyaline bodies and the immunohistochemical spectrum were typical for UES of the liver [Figure 4]. Rhabdomyosarcomatous differentiation was not seen. Based on these findings, we arrived at a final diagnosis of UES of the liver.

This patient is presently on chemotherapy (ifosfamide, doxorubicin and ondansetron) and follow-up imaging (CT scan) has revealed mild reduction in size. Future plans include surgery, if feasible, followed by radiation and postoperative chemotherapy. This is the currently accepted clinical practice.

## Discussion

UES is a highly malignant primitive mesenchymal tumor that occurs predominantly in older children and adolescents, although it has occasionally been reported in adults.[1–5] Although it represents about 9-15% of all hepatic tumors in children, only about 150 cases have been reported in the literature.[1–2]

The histogenesis of this tumor still remains undetermined. Some authors have suggested that it is the malignant counterpart of mesenchymal hamartoma.[1] Several cytogenetic abnormalities, including TP53 mutation, chromosome 19q and 7q, have been investigated to understand the histogenesis of this tumor.[2]

CT scan typically demonstrates a large mass with cystic attenuation, while on MRI, large portions of the mass are hypointense on T1W images and have a high signal intensity on T2W images. Areas of high signal intensity on T1W images and low signal intensity on T2W images may also be seen in some cases due to intratumoral hemorrhage.[1–6] With gadolinium administration, there is mild heterogeneous enhancement with predominant lack of enhancement in most of the tumor, consistent with extensive central necrosis or cystic change. Moon *et al*.[7] have also described the discrepancy of internal architecture on USG and CT scan as one of the important characteristics of UES.

Buetow *et al*.,[3] who have studied the pathological basis of imaging findings in cases of UES, have stated that there is an excellent correlation between the predominantly solid USG appearance and the pathologic findings in these liver tumors, whereas there is discordance between the CT scan/MRI findings and the USG and pathologic analysis.[3] According to the authors, the increased water content within the abundant myxoid stroma of UES accounts for the attenuation lower than that of soft tissue on CT scans and the high signal intensity on T2W MRI images, as seen in our case. Similar findings have also been reported on CT scan and MRI with other myxomatous tumors. UES, in 83% of the cases, appears predominantly solid at gross examination.[3] It is not surprising that MRI is more likely than CT scan to depict areas of hemorrhage, as was seen in our case.

Histopathologically, the tumor has a variable but distinctive sarcomatous appearance. UES is composed of spindle- and stellate-shaped sarcomatous cells, with marked nuclear pleomorphism or multinucleate forms, closely packed in whorls or sheets or scattered loosely in a myxoid ground substance.[2–3] The cell borders are poorly defined. Pleomorphic multinucleated giant cells are relatively frequent.

The differential diagnosis in this 7-year-old child included UES, hepatocellular carcinoma (HCC), complicated hydatid cyst, hematoma and abscess (amebic, bacterial). We considered HCC and EUS, as HCC is the most common primary malignant hepatic tumor in children more than 5 years of age while UES is typically seen in children in the age group of 6-10 years and, along with HCC, accounts for the majority of all malignant hepatic neoplasms in children more than 5 years old. However, HCC was considered less likely as the liver was noncirrhotic, the lesion appeared cystic on CT and the patient did not have elevated α-fetoprotein levels. Complicated hydatid cyst and liver abscess were considered as the patient was febrile and imaging features were suggestive. However, blood culture was negative and did not grow any organism and serology for hydatid was also negative. Although we were not provided with a direct history of trauma, it was considered in the differential list as the history is not always forthcoming and USG features were quite suggestive.

Until the late 1980s, survival was considered poor for patients with UES. However, survival outcome in the early 1990s has improved considerably with the use of multidrug combination chemotherapy, including sarcoma protocols.[6]

Awareness about these discordant and paradoxical USG and CT scan findings in UES of the liver is useful for the differential diagnosis of primary hepatic tumors in childhood.
